# Management of Penicillin Allergy in the Perioperative Setting

**DOI:** 10.3390/antibiotics13020157

**Published:** 2024-02-05

**Authors:** Mary Elizabeth Sexton, Merin Elizabeth Kuruvilla

**Affiliations:** 1 Division of Infectious Diseases, Emory University School of Medicine, Atlanta, GA 30322, USA; 2 Department of Internal Medicine, Emory University School of Medicine, Atlanta, GA 30322, USA; merin.kalangara@emoryhealthcare.org; 3 Novartis Pharmaceuticals, East Hanover, NJ 07936, USA

**Keywords:** penicillin allergy, perioperative antibiotic prophylaxis, beta-lactam cross-reactivity, allergy de-labeling

## Abstract

The selection of perioperative antibiotic prophylaxis is challenging in patients with a history of penicillin allergy; as such, we present a literature review exploring current best practices and the associated supporting evidence, as well as areas for future research. Guidelines recommend the use of alternative agents in patients with an IgE-mediated hypersensitivity reaction, but those alternative agents are associated with worse outcomes, including an increased risk of surgical site infection, and higher cost. More recent data suggest that the risk of cross-reactivity between penicillins and cephalosporins, particularly cefazolin, is extremely low, and that cefazolin can be used safely in most penicillin-allergic patients. Studies have therefore explored how best to implement first-line cefazolin use in patients with a penicillin allergy label. A variety of interventions, including preoperative allergy de-labeling with incorporation of penicillin skin testing, use of patient risk-stratification questionnaires, and utilization of clinician algorithms to guide antibiotic selection intraoperatively, have all been shown to significantly increase cefazolin utilization without a corresponding increase in adverse events. Further studies are needed to clarify the most effective interventions and implementation strategies, as well as to evaluate whether patients with severe delayed hypersensitivity reactions to penicillin should continue to be excluded from receipt of other beta-lactams.

## 1. Introduction

Perioperative antibiotic prophylaxis is an essential component of surgical site infection (SSI) prevention in many types of procedures [1,2]. The current consensus guidelines from the Infectious Diseases Society of America (IDSA), the Surgical Infection Society (SIS), the Society for Healthcare Epidemiology of America (SHEA), and the American Society of Health-System Pharmacists (ASHP) recommend cefazolin utilization in most procedures, with alternative recommendations for some surgical patients with a penicillin allergy label [3]. Those guidelines are undergoing an update, but the existing version advises avoidance of beta-lactam antibiotics in patients with an IgE-mediated penicillin allergy, including any bronchospasm, urticarial rash, or other manifestations of an anaphylactic reaction, and patients with a severe cutaneous adverse reaction (SCAR) to any beta-lactam (e.g., Stevens–Johnson syndrome). Vancomycin (either alone or with the addition of gentamicin, aztreonam, or a fluoroquinolone in cases where Gram-negative coverage would be indicated) and clindamycin are the most commonly recommended alternatives in patients with a life-threatening penicillin allergy, although the guideline authors acknowledge that many patients with non-life-threatening allergies will also receive these alternatives because of a lack of clarity about patient allergy histories [3]. This use of alternative agents in patients with a history of penicillin allergy comes with significant consequences, including an increased risk of SSIs and adverse drug reactions, and so efforts are ongoing to understand the circumstances in which cefazolin use is safe in penicillin-allergic surgical patients, and the methods by which changes to allergy evaluation protocols can best be operationalized. We have therefore reviewed the existing literature regarding SSI risk in penicillin-allergic patients, risks and benefits of the use of cefazolin and alternative agents for perioperative prophylaxis, safety of cefazolin in patients with a penicillin allergy label, methods for increasing utilization of cefazolin when it is safe to do so, and best practices for implementing those methods.

## 2. SSI Risk in Penicillin-Allergic Patients

Multiple studies have identified an increased SSI risk in penicillin-allergic patients with the use of beta-lactam alternatives for perioperative prophylaxis, which is present across multiple procedure types, including orthopedic surgeries, oral and maxillofacial surgeries, obstetric procedures and gynecologic surgeries, colorectal surgeries, and cardiac surgeries [4,5,6,7,8,9]. Blumenthal et al. found that penicillin-allergic patients had an adjusted odds ratio of 1.51 (95% CI 1.02–2.22) for developing an SSI following a surgical procedure compared to those without a reported allergy, which they attributed to the use of antibiotics other than cefazolin in these patients [9]. Novell et al. directly compared patients who received cefazolin prior to total knee arthroplasty to those who received second-line clindamycin and/or vancomycin, and found a higher rate of any SSI and of prosthetic joint infections in the second-line group [5]. The etiology of this SSI risk associated with use of second-line antibiotics may be multifactorial, including the antibiotic spectrum of coverage and challenges with timing and dosing [10].

## 3. Challenges with the Use of Beta-Lactam Alternatives for Perioperative Prophylaxis

### 3.1. Spectrum of Coverage

Increasing clindamycin resistance has been documented in community-onset bacterial infections, including those caused by skin flora that are a common etiology of SSIs [7,11]. An evaluation of patients with orofacial infections from 2009 to 2014 found that 32% of *Streptococcus viridans* isolates and 23% of *Staphylococcus* species isolates were resistant to clindamycin, compared to 13.7% and 10.5%, respectively, in isolates from 1997 to 2003 [11]. While vancomycin has Gram-positive coverage, it is not the first-line therapy for methicillin-susceptible *Staphylococcus aureus* infections [10], and a large Australian study found that patients who received vancomycin rather than a beta-lactam antibiotic for surgical prophylaxis had a significantly increased likelihood of an SSI due to MSSA (OR 2.79; 95% CI 1.60–4.87) [12]. It also lacks Gram-negative coverage compared to cefazolin and has therefore been linked to an increased incidence of Gram-negative SSIs when it is used as a single agent in penicillin-allergic patients [10,13].

### 3.2. Administration Logistics

Vancomycin additionally needs to be administered over at least an hour, which can create challenges with ensuring that it is given sufficiently early pre-procedure such that levels are appropriate at the time of incision. Blumenthal et al. found that 97.5% of patients receiving vancomycin had their dose administration started outside of the 60–120 min window pre-procedure that was being targeted per hospital protocol, with the median start time only 24 min before incision [9]. A similar issue may be reflected in the findings of another study of more than 800 patients undergoing caesarian section, where 9.2% of patients with a penicillin allergy did not receive their perioperative antibiotic within an hour of the case start, compared to only 6.9% without a documented allergy (*p* = 0.019) [14].

Both initial dosing and intraoperative re-dosing can also make the use of vancomycin prophylaxis challenging. A study of patients undergoing total joint arthroplasty found that 62% of patients receiving vancomycin prophylaxis were given an underdose of vancomycin based on their weight. While this underdosing was not associated with an increased risk of SSI overall compared to those with adequate dosing, postoperative MRSA infections were seen among underdosed patients but not among those who received adequate dosing [15]. The patients receiving vancomycin at any dosing level were 50% more likely to have a postoperative prosthetic joint infection than those receiving cefazolin, which the authors hypothesized might be the result of either differences in the spectrum of coverage with respect to Gram-negative pathogens, or to declining vancomycin levels over the course of cases [15]. Prior studies had identified that vancomycin levels can fall to subtherapeutic levels prior to surgical closure when vancomycin is underdosed or the case is lengthy [16]; in this study the authors estimated that on average their patients would have had a subtherapeutic vancomycin level after 3 h [15].

Vancomycin also may need to be given in combination with another agent in cases where Gram-negative coverage is needed, and compliance with multi-drug perioperative regimens may be decreased compared to single-drug regimens. For example, Burzyńska et al. found that compliance with appropriate timing and dosing of cefazolin administration alone was almost ten times greater than compliance with cefazolin plus a second antibiotic [17].

### 3.3. Adverse Drug Reactions

The use of alternative antibiotic agents in penicillin-allergic patients has also been linked to a higher rate of adverse drug reactions. Vancomycin has been associated with nephrotoxicity, as has gentamicin, which is sometimes given in combination for Gram-negative prophylaxis. A retrospective large cohort study of surgical patients at Veterans Affairs (VA) Hospitals from 2008 to 2013 found that patients receiving vancomycin for perioperative prophylaxis were 12–30% more likely to develop acute kidney injury (AKI) than patients receiving a beta-lactam antibiotic [18]. A systemic review and meta-analysis of 11 studies found that patients receiving perioperative gentamicin for orthopedic surgeries had a risk ratio of 2.99 (95% CI 1.84–4.88) for developing an AKI following surgery [19]. There are also case reports of ototoxicity with single-dose gentamicin administration, including one where perioperative gentamicin in a patient with an underlying history of Meniere’s disease led to an inability to drive, work, or walk without help secondary to vestibular damage [20].

Overall, the risk of any adverse drug event (including allergic reactions) appears to be higher with alternative agents than with cefazolin. In one study of ~17,000 pediatric surgical procedures, the rates of adverse drug events in patients receiving cefazolin were similar regardless of penicillin allergy history (0.75–1.04%), while 3.3% of patients receiving vancomycin had an adverse event [21].

### 3.4. Healthcare-Associated Infections

There is also concern for an increased risk of healthcare-associated infections with utilization of beta-lactam alternatives. A study that retrospectively assessed patients in the UK with a documented penicillin allergy and matched controls with no allergy over a mean of 6 years found that patients with a penicillin allergy had an adjusted hazard ratio of 1.69 (95% CI 1.51–1.90) for developing an MRSA infection (with 55% of that risk attributed to use of non-beta-lactam antibiotics) and of 1.26 (95% CI 1.12–1.40) for being diagnosed with *C. difficile* colitis (with 35% of the risk attributed to non-beta-lactam antibiotics) [22]. An increased risk of *C. difficile* may be of particular concern with use of clindamycin for perioperative prophylaxis, as one study identified an alteration in the gut microbiome for up to 4 months following clindamycin administration [23]. Clindamycin use was also associated with postoperative urinary tract infections in a bariatric surgery population in multivariable analysis (*p* ≤ 0.0001) [24].

### 3.5. Summary

There are challenges with respect to coverage spectrum, timing of administration relative to incision, adverse drug reactions, and increased risk of SSI and other healthcare-associated infections when non-beta-lactam alternative agents are used for perioperative prophylaxis (see Table 1).

## 4. Beta-Lactam Allergy and Cross Reactivity

All of the above challenges associated with the use of alternative agents are compounded by data suggesting that most reported penicillin allergies are either not true allergies or not life-threatening, and that the cross-reactivity between penicillin and cefazolin is negligible [25,26]. There is therefore an ongoing push toward use of cefazolin in these patients whenever possible.

### 4.1. Penicillin Allergy and Cross-Reactivity with Cephalosporins

The term “penicillin allergy label” (PAL) refers to an unconfirmed penicillin allergy, with a prevalence of ~10% in the US population [27]. Cefazolin is often avoided in surgical patients with a PAL for fear of cross-reactivity, despite true penicillin hypersensitivity being rare (<5%) in patients with a PAL [27], and an extremely low likelihood of cross-reactivity between penicillins and cephalosporins (1–2%) [28].

**Figure 1:**
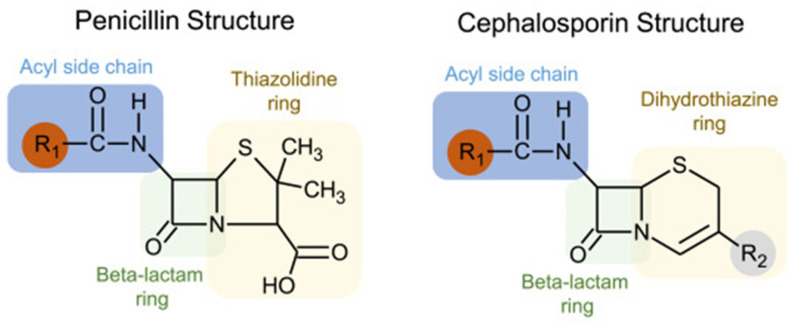
Structural basis of cross-reactivity between penicillins and cephalosporins. Penicillins and cephalosporins share common structures: (1) a beta-lactam ring, shown in green; (2) side chains or R groups, with the R1 location shown in red. Cross-reactivity is largely based on R1 side chains, and cefazolin has a unique R1 side chain that is not shared with other beta-lactams. (Reproduced with permission from Khan DA et al., “Drug allergy: A 2022 practice parameter update,” *Journal of Allergy and Clinical Immunology* 2022; Vol 150(6): 1333–1393 [30]).

Understanding the immunogenic components of beta-lactams may clarify why the cross-reactivity rate between penicillin and cephalosporins is negligible. While all beta-lactams share a core beta-lactam ring, penicillin has a five-membered thiazolidine ring that is retained following its breakdown, which forms the immunogenic component. On the other hand, cephalosporins have a six-membered dihydrothiazine beta-lactam ring that is rapidly degraded. The primary immunogenic components of cephalosporins are instead formed by their R1 (and occasionally R2) side chains [29]. Figure 1 outlines the structural basis of cross-reactivity between penicillins and cephalosporins, which largely derives from similarities in the R1 side chain.

Cephalosporin allergy is thus mediated by the R1 and R2 side chains and is unrelated to the shared core beta-lactam ring, which likely explains the low cross-reactivity rate with penicillin. Tolerance to cephalosporins in patients with a PAL can usually be predicted based on the degree of similarity between the R1 side chains of the cephalosporin and penicillin [29]. A meta-analysis evaluating the risk of cross-reactivity examined cephalosporin skin testing results in patients with a proven penicillin allergy and showed a low risk of reactivity to low-similarity cephalosporins [28].

### 4.2. Differentiation of Cefazolin from Other Beta-Lactams

A low likelihood of cross-reactivity is especially true for cefazolin [29]. Cefazolin possesses a unique R1 side chain containing a heterocyclic ring that is structurally distinct from other beta-lactams. Several studies have confirmed side-chain specificity in cefazolin hypersensitivity, which appears to be selective, with patients allergic to cefazolin having good tolerance of other β-lactams [31]. Cefazolin thus should be safe for administration in most patients with penicillin or other cephalosporin allergies.

In one recent meta-analysis of 77 studies, the frequency of concomitant penicillin and cefazolin allergy labels in surgical patients was only 0.7%, and an even lower incidence (0.1%) was reported for surgical patients whose penicillin allergy was unconfirmed [32]. Although the risk of cefazolin allergy was indeed greater in patients with confirmed penicillin allergy, the authors suggested that this greater risk may reflect the patient population with drug allergies having a greater incidence of concomitant drug hypersensitivities, instead of being entirely specific to cefazolin. Patients with confirmed penicillin allergy have a greater tendency to develop de novo hypersensitivity reactions to other drugs including cephalosporins, not due to cross-reactivity [32]. These results should facilitate the utilization of perioperative cefazolin use in penicillin-allergic patients, particularly when this allergy history is unconfirmed.

The negligible cross-reactivity between cefazolin and penicillins is reflected in the drug allergy practice parameters issued jointly by the American Academy of Allergy, Asthma, and Immunology (AAAAI) and the American College of Asthma, Allergy and Immunology (ACAAI). Their 2022 updated guidelines recommend the administration of cefazolin to most penicillin-allergic patients, including both those with a history of IgE-mediated reactions including anaphylaxis, and those with a non-anaphylactic reaction to penicillin [30]. However, this recommendation does not apply to patients with histories of severe delayed, presumably T-cell-mediated, immune reactions. These include SCARs such as Stevens–Johnson syndrome/toxic epidermal necrolysis and DRESS syndrome, as well as organ-specific reactions such drug-induced liver injury [30].

## 5. Evidence Supporting Safety of Cefazolin Use in Most Patients with PALs

In recognition of the profound impact of second-line antibiotic use in patients with a PAL, several studies have evaluated methodologies to optimize perioperative antibiotic selection. All of these studies, while using varied interventions, have consistently demonstrated uneventful administration of cefazolin to patients with a PAL.

The safety of cefazolin use in surgical patients with a PAL has been described in several retrospective studies. In one review of 413 surgical patients with a PAL who received preoperative cefazolin, a possible allergic reaction was noted in only one case [33]. Similarly, in another retrospective institutional study of 549 procedures in patients with a PAL who were given cefazolin prophylaxis, there were no allergic symptoms related to its use [34]. More recently, 46% of 143 patients with a documented PAL were administered cefazolin in a review of 2451 joint arthroplasties. Only one patient with a documented PAL who received cefazolin had an allergic reaction, which was non-severe and resolved without additional treatment; there was no difference in the overall reports of allergic reactions in patients with and without a PAL [35]. Finally, Beltran et al. reported only one allergic reaction to cefazolin out of 127 pediatric patients (0.8%) with IgE- and non-IgE-mediated penicillin allergies [36].

## 6. Options for Management of Surgical Patients with a PAL

Based on this emerging evidence, surgical programs have explored how best to design and implement new guidelines and educate proceduralists, in order to encourage cefazolin use in appropriate patients with a PAL. Studies have evaluated opportunities to clear allergy labels at pre-procedure appointments, as well as algorithms for assessing cefazolin safety at the time of surgery.

### 6.1. Allergy De-Labeling Prior to Elective Surgery

One strategy to promote cefazolin use in surgical patients with a PAL has been through preliminary penicillin skin testing (PST). Several recent publications in orthopedic surgery describe the effectiveness of allergy referral programs for penicillin skin testing to optimize perioperative antibiotic prophylaxis [37,38]. In these cohorts, 97–99% of patients had their penicillin allergy de-labeled, with an associated decreased risk of subsequent prosthetic joint infection compared to those who still received a second-line antibiotic. In another cohort of patients with a PAL undergoing cardiac surgery, preoperative penicillin allergy testing increased the use of first-line antibiotics from 38% to 92% (*p* < 0.001) [39]. Moussa et al. similarly evaluated an algorithm for referring penicillin-allergic patients for allergy evaluation at their preoperative appointment; those deemed likely to have a true allergy underwent PST followed by a single- or graded-dose oral challenge to assess for the ability to de-label them. In total, 85% of the patients who required perioperative prophylaxis following negative PST and oral challenge results received cefazolin [40]. More recent institutional studies have continued to evaluate and recommend the inclusion of penicillin skin testing in the preoperative workflow. One study of patients undergoing elective urogynecologic surgery found that 64% of patients referred for allergy de-labeling participated in that evaluation, with a subsequent increase in perioperative cefazolin use from 48% to 76% [41]. Another study used a tiered approach where patients were immediately de-labeled in the preoperative clinic if their described reaction was non-allergic (e.g., development of thrush), with 72.4% of the patients who qualified agreeing to de-labeling. Other patients were referred for direct oral challenge, PST, or detailed allergist evaluation depending on the severity of their reaction history [42].

### 6.2. Patient Screening Questionnaires as Part of Preoperative Evaluation

Other institutions and health systems have employed different clinical decision support tools to optimize cefazolin administration for surgical prophylaxis. In one study, a straightforward approach of incorporating a structured allergy history into the preoperative assessment of PAL patients, with subsequent exclusion of those reporting anaphylaxis and severe reactions such as SCAR or organ injury, decreased the use of alternative antibiotics from 81.9% to 55.9% and increased cefazolin use from 18% to 57% [43]. The orthopedic surgery group at the University of Rochester implemented a multimodal stewardship intervention that included assessment of the penicillin allergy history at the preoperative visit [44]. Patients whose allergies were not severe (including those with hives or localized swelling) were ordered to receive cefazolin preoperatively, and those identified as severe (including anaphylaxis) had an option for referral to an allergist for preoperative assessment. Four patients were ultimately referred, with all four cleared to receive cefazolin at the time of surgery, and overall perioperative antibiotic use classified as appropriate in penicillin-allergic patients increased from 54% to 91% after the stewardship interventions [44].

### 6.3. Utilization of Clinical Decision Support Tools at the Time of Surgery

While the above methods were linked to significant improvements in perioperative antibiotic use, not all patients are assessed at a preoperative clinic visit. Kuruvilla et al. therefore described the outcomes of using a streamlined algorithm for evaluation of PALs in preoperative patients at the time of surgery. The algorithm encouraged use of cefazolin or cefuroxime in almost all patients with a history of penicillin allergy, including those with anaphylaxis, and with only those with severe delayed reactions excluded. The percentage of patients receiving a first-line cephalosporin increased from 22% at baseline to >80% following algorithm implementation, without any hypersensitivity reactions attributable to cefazolin use [45]. A similar allergy risk stratification protocol (ACCEPT) was implemented in a Canadian hospital, with use in both preoperative clinics and at the time of surgery for patients not previously assessed. The algorithm recommended utilization of cefazolin in all patients who did not have a history of a SCAR with beta-lactam administration or a documented allergy to cefazolin. Implementation significantly increased cefazolin use among surgical patients with a reported beta-lactam allergy (80% post-intervention vs. 40% pre-intervention; *p* = 0.002) [46].

Rather than using an algorithm for PAL assessment by providers, another study evaluated implementation of an institutional policy that defaulted to cefazolin use for surgical prophylaxis in almost all patients with a PAL. The hospital made modifications to the perioperative order set such that alternative antibiotics were not listed for penicillin allergies other than a history of SCAR; the study found no association between a PAL and a reaction to cefazolin [47].

Collins et al. incorporated a chart showing the likelihood of cross-reactivity between penicillins and cephalosporins based on side-chain similarity into the preoperative workflow as part of an allergy assessment to guide surgical prophylaxis in patients with a PAL [48]. Significantly fewer patients received a non-beta-lactam agent post-intervention (from 84.9% down to 15.1%; *p* < 0.001) without a difference in allergic reactions. Of note, 8 patients out of 1089 in the intervention cohort who received first line beta-lactam prophylaxis had historical types II–IV hypersensitivity reactions (e.g., SCARs, drug fever, hemolytic anemia, or serum sickness), and there were no reported adverse reactions [48].

## 7. Efficacy and Cost Data

The increased rates of cefazolin administration in all the above studies were not accompanied by a corresponding increase in the rate of allergic reactions, but did lead to improvements in patient outcomes. For example, Kwiatkowski et al. found that a pharmacist-led intervention to evaluate and address PAL labels prior to elective surgery led to both an increase in cefazolin use (from 28% in the control group to 65% in the intervention group) and a lower rate of SSIs (none reported in the group who were part of the intervention versus 10% in the control group) [49]. In another study of orthopedic procedures in which 46% of patients with a PAL received cefazolin, the rate of prosthetic joint infection in patients receiving an alternative agent was almost double that of the group receiving cefazolin [35].

In addition, the use of cefazolin has also been demonstrated to have a cost benefit. Stonerock et al. identified an antibiotic cost savings of 50–80% based on claims data when cefazolin was used rather than a second-line alternative [50]. Sexton et al. similarly identified an almost 50% decrease in the cost of antibiotics per penicillin-allergic patient, following an intervention that increased cephalosporin administration from ~34% to more than 80% in that group [51].

## 8. Implementation of Perioperative PAL Interventions: Challenges and Opportunities

While the algorithms and protocols discussed above have been shown to be effective, the associated studies have also identified the importance of considering what is necessary for implementation, including acceptance from healthcare providers and staff, and evaluation of opportunities to leverage an electronic medical record (EMR). There are also multiple different types of interventions that an institution could implement detailed in these studies, with advantages and disadvantages to the various options that should be assessed.

### 8.1. Consensus Building and Provider Education

Because use of cefazolin in the majority of patients with a PAL is a deviation from some consensus guidelines [3] and typical clinical practice, multiple studies have identified provider education as an important component to these interventions [52]. A survey of anesthesiologists in the UK found that only 40% were comfortable giving penicillin to a patient where they believed the allergy label was likely inaccurate, and only 47% were comfortable giving penicillin to a patient who had been de-labeled by an allergist [53]. The anesthesiologists cited beliefs that de-labeling required skin testing and a desire to have formal guidance from their healthcare facilities supporting the use of penicillin in patients with a PAL prior to proceeding with its use [53]. Therefore, both education interventions and leadership support are critical [52]. As a result, multidisciplinary involvement in provider education is a theme, with participation from a combination of pharmacists, surgeons, anesthesiologists, infectious disease physicians, and allergists noted as successful in multiple studies [46,51,54]. Grand rounds and other educational sessions have been utilized to allow allergists to present the associated safety data [51,55]. Subsequent involvement of proceduralists as sponsors/champions of these interventions may help to garner further support. For example, Jones et al. relied on an orthopedic surgeon serving as the project champion as part of their multipronged intervention that increased appropriate use of antibiotics by more than 35 percentage points in penicillin-allergic patients undergoing total joint arthroplasty [44].

### 8.2. Use of the Electronic Medical Record

An EMR can both pose challenges to and help with implementation of algorithms for patients with a PAL. Many EMRs fire alerts for allergy cross-reactivity, including for use of cephalosporins in patients who have a documented history of penicillin allergy [52]. Especially in the setting of the hesitancy some providers may have around giving cefazolin to a patient with a PAL, as evidenced by the results of the anesthesiologist survey described above, these alerts could hurt efforts to expand appropriate cephalosporin use. Macy et al. therefore evaluated an intervention to turn off alerts about cephalosporin prescribing in penicillin-allergic patients at one hospital, while leaving them active at another hospital. Cephalosporin use increased from 17.9% to 27.0% of antibiotic courses at the hospital with the alerts turned off, while it remained statistically unchanged at the hospital with the alerts still active [56].

Other studies have leveraged the EMR for assistance. In Isserman et al.’s study evaluating multiple interventions to increase cefazolin use in penicillin-allergic pediatric patients, they arranged for automated educational e-mails to be sent to clinicians if they ordered clindamycin prophylaxis for a patient whose penicillin allergy was documented as non-severe in the EMR [55]. VanderVelde et al. used EMR messaging to communicate both with clinicians and pharmacies about a patient’s history of tolerating penicillin, and with patients themselves to obtain more details about their allergy history [54].

### 8.3. Advantages and Disadvantages of Potential Workflows

While the studies described above all demonstrated an increase in cefazolin utilization in patients with a PAL, those results were obtained using multiple different interventions. The majority of these intervention types have not been compared head-to-head, and so selecting which intervention(s) to pursue at an institution may need to take a variety of factors into account.

One of the first decision points is whether to attempt to de-label patients with respect to their penicillin allergy, or whether to focus on algorithm development to give cefazolin safely to patients with a PAL. De-labeling patients comes with some advantages, such as addressing the bigger issue of erroneous PALs to optimize antimicrobial stewardship, and potentially increasing both clinician and patient comfort with beta-lactam use. In Savic et al.’s survey of anesthesiologists, there were significant concerns documented about giving penicillin to a patient with a PAL without clear backing from their institution [53]; these types of concerns might be alleviated if the allergy label had already been removed. How de-labeling is undertaken may also matter, as more than 25% of patients in a study evaluating removal of the PAL for a history inconsistent with true allergy refused de-labeling without undergoing allergy testing [42].

Some studies relied on PST, either alone or in combination with oral challenge, to perform de-labeling. However, the utility of PST is confined to patients with a history of IgE-mediated penicillin allergy, and PST has been negative in studies of patients with cefazolin-induced hypersensitivity, suggesting that this preliminary step may not rule out potential reactions to cefazolin [31,57]. This was corroborated in a recent study of 83 patients with confirmed perioperative cefazolin-induced allergic reactions with no previous history of penicillin allergy [58]. While seven patients had positive or equivocal PSTs, these patients had uneventful drug provocation tests. Recognition of this lack of cross-reaction is reflected in the updated drug allergy practice guidelines, as per which formal penicillin allergy testing is no longer considered necessary to promote cefazolin use in surgical patients including in patients with anaphylaxis [30].

Other challenges with de-labeling include the need for preoperative coordination and the resources and cost involved. In Vaisman et al.’s study, only 50% of surgical patients had a preoperative visit where their allergy questionnaire could be administered [43], suggesting that relying on preoperative clinic evaluation to assess allergy history and refer for additional testing/de-labeling may miss significant numbers of patients. Preoperative allergy assessment and/or de-labeling also involve a significant financial and time outlay. Liu et al. performed an evaluation of the cost of implementing PST relative to the benefit, and they found that the degree to which performing PST made financial sense was dependent on the rates of *C. difficile* infection and SSIs at an institution [59]. PST supplies and training are also not always available—less than 50% of health systems have access to PST, making this approach less likely to be generalizable [60].

**Figure 2:**
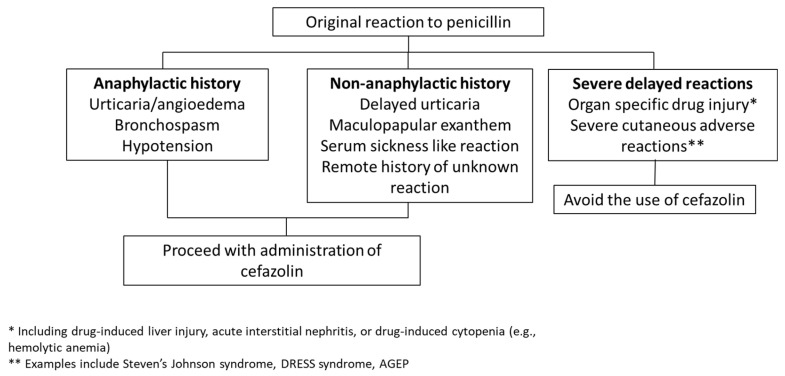
Proposed algorithm for cefazolin use in patients with a history of penicillin allergy. One approach to penicillin-allergic patients is to administer perioperative cefazolin except in cases where the allergy involved a SCAR or organ injury, using an algorithm based on Khan et al.’s 2022 practice parameter [30], similar to those utilized by Kuruvilla et al. [45] and Lam et al. [46] without adverse reactions reported.

Direct cefazolin administration without preliminary skin testing eliminates these logistical barriers and therefore may provide a more expeditious way to manage low-risk PAL patients. The algorithms used in studies have differed, with some including penicillin anaphylaxis history in a list of contraindications to cefazolin use and others focusing only on a history of SCAR or organ dysfunction as contraindications. Use of the latter more permissive criteria has been associated with larger improvements in cefazolin utilization without identified safety concerns [45,46], and so may be preferable for easier implementation. Figure 2 outlines a suggested approach to cefazolin use in surgical patients with reported penicillin allergy in that setting. Direct use of cefazolin without de-labeling likely requires additional education to ensure clinician and patient comfort, and some of the EMR interventions discussed above may therefore be beneficial if selecting that route.

## 9. Future Directions

One unanswered question remains the extent to which cefazolin cross-reacts with penicillins in the population with histories of severe delayed reactions such as SCARs and organ-specific drug injury. Beta-lactam cross-reactivity in patients with SCARs appears to be R1 side-chain specific, and reports of SCARs induced by two separate classes of beta-lactams are infrequent; however, the data are incomplete [30]. Currently, avoidance of all beta-lactams is recommended in patients with a history of SCAR that is deemed highly likely to be related to a beta-lactam, butaccumulating data from small case series suggest tolerance of cephalosporins in patients with severe T-cell-mediated reactions to penicillins [61]. Also, tolerance of cefazolin specifically has been described in five patients with histories of nafcillin-induced organ-specific drug injury (immune-mediated nephritis/hepatitis) or serum sickness [62]. However, larger studies are needed to determine the safety of alternative beta-lactam use in patients with a history of SCAR secondary to a specific beta-lactam.

## 10. Conclusions

In view of the significant risks associated with administration of second-line antibiotics for perioperative prophylaxis, efforts are ongoing to optimize the use of cefazolin in surgical patients with PALs. Cefazolin use is safe in most PAL scenarios and is now recommended for routine administration without preemptive testing for penicillin tolerance in updated drug allergy practice parameters. However, there remains a discrepancy with national clinical practice guidelines for surgical prophylaxis, which need modification to provide standardized antibiotic recommendations that align with our current understanding. In addition, educational efforts are required to reinforce this reassuring data to anesthesiology and surgical providers. Given the known benefits of appropriate perioperative antibiotic choice, additional research should focus on the most expedient way to accomplish this goal, whether using clinical decision support tools or removing cross-reactivity warnings for structurally dissimilar beta-lactams in patients with low-risk PALs, in order to optimize perioperative cefazolin use.

## Figures and Tables

**Table 1:** Comparison of perioperative prophylaxis agents for patients with a history of penicillin allergy.

Perioperative Prophylaxis Option	Advantages of Utilization	Challenges with Utilization
Cefazolin	First-line agent per guidelines Coverage spectrum suited to skin flora Can be administered rapidly	Avoidance recommended in those with severe delayed hypersensitivity reactions to beta-lactam antibiotics Does not provide MRSA coverage, so an additional agent is needed with risk factors
Vancomycin	Provides MRSA coverage in high-risk cases Safe in patients with severe delayed hypersensitivity reactions to beta-lactam antibiotics	Increased risk of SSIs with use, including MSSA infections and Gram-negative infections, because of coverage spectrum Needs to be administered over at least an hour, making appropriate timing relative to surgical incision difficult Dosing is weight-based and redosing requires monitoring of drug levels Increased risk of adverse drug events, including nephrotoxicity
Clindamycin	May provide MRSA coverage Safe in patients with severe delayed hypersensitivity reactions to beta-lactam antibiotics	Increasing resistance to *Staphylococcal* species and *Streptococcal* species documented Increased risk of healthcare-associated infections, including *C. difficile* infection and post-surgical UTI, has been documented Gut microbiome disruption

Cefazolin remains the first-line agent for perioperative prophylaxis in most surgical cases, as a result of the challenges associated with use of non-beta-lactam antibiotic alternatives, including vancomycin and clindamycin [3,7,9,10,11,12,13,14,15,16,18,21,22,23,24]. MRSA = Methicillin-resistant *Staphylococcus aureus*, SSIs = surgical site infections; MSSA = Methicillin-sensitive *Staphylococcus aureus*; UTI = urinary tract infection.
